# Manipulation of an Innate Escape Response in *Drosophila*: Photoexcitation of *acj6* Neurons Induces the Escape Response

**DOI:** 10.1371/journal.pone.0005100

**Published:** 2009-04-02

**Authors:** Gregor Zimmermann, Li-ping Wang, Alexander G. Vaughan, Devanand S. Manoli, Feng Zhang, Karl Deisseroth, Bruce S. Baker, Matthew P. Scott

**Affiliations:** 1 Departments of Developmental Biology, Genetics, and Bioengineering, Stanford University, Stanford, California, United States of America; 2 Departments of Bioengineering and Psychiatry, Stanford University, Stanford, California, United States of America; 3 Department of Biological Sciences, Stanford University, Stanford, California, United States of America; Yale School of Medicine, United States of America

## Abstract

**Background:**

The genetic analysis of behavior in *Drosophila melanogaster* has linked genes controlling neuronal connectivity and physiology to specific neuronal circuits underlying a variety of innate behaviors. We investigated the circuitry underlying the adult startle response, using photoexcitation of neurons that produce the abnormal chemosensory jump 6 (*acj6*) transcription factor. This transcription factor has previously been shown to play a role in neuronal pathfinding and neurotransmitter modality, but the role of *acj6* neurons in the adult startle response was largely unknown.

**Principal Findings:**

We show that the activity of these neurons is necessary for a wild-type startle response and that excitation is sufficient to generate a synthetic escape response. Further, we show that this synthetic response is still sensitive to the dose of *acj6* suggesting that that *acj6* mutation alters neuronal activity as well as connectivity and neurotransmitter production.

**Results/Significance:**

These results extend the understanding of the role of *acj6* and of the adult startle response in general. They also demonstrate the usefulness of activity-dependent characterization of neuronal circuits underlying innate behaviors in *Drosophila*, and the utility of integrating genetic analysis into modern circuit analysis techniques.

## Introduction

An important goal of neurobiology is to understand the relationship between specific neural circuits and corresponding motor, sensory, and behavioral outcomes. Efforts to ablate or stimulate neural and muscle cells to map their functions have a long history. In 1709-10 Petit and Mistichelli determined that damage to one side of the brain was linked to lost control of movement on the opposite side of the body [1]. In 1746, the French physicist Nollet lined up a chain of 200 monks and sent an electrical shock through the line [2], while in 1791, Galvani used lightning as a power source for stimulating contraction of frog legs [3]. In more recent times, neuroscientists have used more precise stimulation and ablation experiments to map the neural control of behavioral processes. Functional mapping and ablation studies have been successfully used to understand the neural basis of processes as diverse as bird song acquisition and visual perception. Similarly, stimulation of defined neural populations has been instrumental in mapping functional connections, as well as probing the roles of perception in behaving animals, from fly to primate [4], [5].

Many electrophysiological studies have relied primarily upon neuroanatomical localization of electrodes to stimulate neural populations, with the precise localization of the stimulus providing specificity. This has been an enormously valuable approach but has limitations, particularly when the experimental animal has a small brain and small neurons or when the behaviors under study require substantial movement. Obstacles to this approach include the need for precise temporal stimulation of functionally related neurons at dispersed locations.

Targeting stimulation to genetically identified neurons offers an alternative approach to the analysis of behavioral circuitry. In particular, neurogenetic approaches to the analysis of behavior have recently been used to identify the neural circuitries underlying several innate behavioral programs. These efforts have identified genes that are expressed in the neurons comprising substantial parts of the circuitry for particular behavioral programs (reviewed in [6]). The specific expression patterns of the genes allow the precise and reproducible identification of key neural populations related to particular behaviors. In addition the transcription control elements of the genes can be used for molecular genetic engineering, targeting the expression of proteins that can be used to manipulate neuronal activity.

Because of its sophisticated genetics and neurobiology, *Drosophila* provides an excellent system in which to integrate physiologic and neurogenetic approaches to the analysis of behavioral circuits. Extensive studies of gene regulation have provided a battery of tools for activating production of a chosen protein in sets of cells with good spatial and temporal specificity. By producing proteins that respond to general, controllable stimuli, for example temperature or light, in specific neurons, exposure of the whole organism to the stimulus influences only certain cells. In this way genetically encoded “switches” produced in specific neural populations can be used to induced or influence fly behavior [7], [8], [9].

Here we employ targeted expression of the *Channelrhodopsin-2 (chr2)* gene in genetically identified neural populations to induce specific innate behaviors via photoactivation of channel function. Channelrhodospin-2 (ChR2) was identified as a light-activated cation-selective ion channel that generates photocurrents in green algae (*Chlamydomonas reinhardtii*) [10]. Soon after the initial introduction and engineering of ChR2 for mammalian neurons [11], it was shown that the expression of ChR2 in a variety of organisms allows for the light-induced depolarization of excitable cells in response to blue (470–490 nm) light [8], [12], [13].

As ChR2 is still a new tool, it is important to test its ability to either prevent or enhance the activities of excitable cells *in vivo*, and to explore its usefulness in altering motor and behavioral functions. In our studies we have tested the temporal precision and reliability of ChR2 in larval neurons and muscle. We demonstrate that pan-neuronal photoexcitation in larva causes contractile paralysis. Our single-unit recordings show that ChR2 stimulation offers precise temporal control of neuronal activity as well as depolarization and contraction of myofibers.

We have applied the photostimulation approach to a genetically specified behavioral circuit. We demonstrate that the activity of *acj6* neurons is necessary for the innate startle response, and that silencing these neurons phenocopies an *acj6* mutation. Photoexcitation of *acj6* neurons induces a synthetic escape response, for which *acj6* expression is itself necessary. Suppressing the photoexcitation of cholinergic *acj6* neurons reduces the escape behavior, showing the importance of cholinergic neurons for the response.

These studies further validate the use of ChR2 in the analysis of neural circuits, show with electrophysiological detail the effect of the stimulation, and apply the approach to the analysis of a behavioral circuit through dual manipulation of neuronal activity and gene function.

## Results

### Optical control of larval behavior using Channelrhodopsin-2

**Figure 1:**
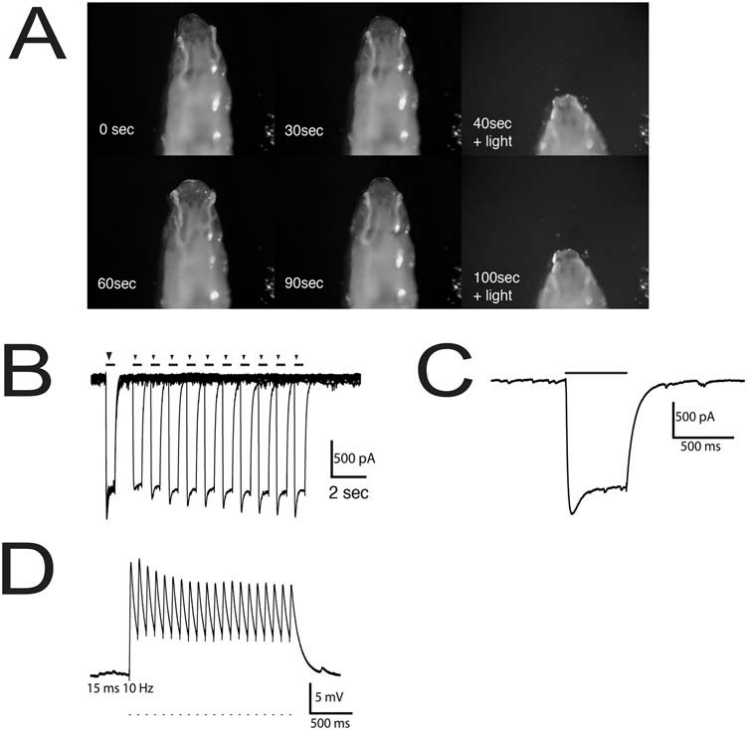
Optical control of muscle depolarization and contraction in *Drosophila* larvae. (A) CO_2_-anesthetized L3 larvae producing ChR2 in muscles (*twi*-*GAL4*; 24B-*GAL4*) display a marked head retraction in response to light stimulation. The larva is immobile during dark periods (0–30 sec, 60–90 sec) but undergoes a rapid contraction in response to brief periods (2–3 second-long light periods at the indicated times) of illumination with blue light (0.2 mW/mm^2^). Moving larvae of the same genotype show complete motion arrest in response to illumination (see Movie S1). Animals were raised in vials supplemented with all-trans retinal. (B–D) One-electrode recordings from larval muscle 6 (for a map of muscle fibers see [31]). (B) Inward current in voltage-clamped muscle fibers, evoked by pairs of 0.5 second light pulses. Several such pairs are overlaid, with a delay varying from 1 to 10 seconds; the first pulse marked by a large arrowhead, and the second pulse from each pair is marked by a small arrowhead. (C) Enlarged view of inward current generated by a single 0.5 sec light pulse. (D) Voltage traces showing membrane depolarization in a current-clamped muscle evoked by 15 ms light pulses delivered at 10 Hz.

We generated transgenic *Drosophila* fly lines that produce a ChR2-YFP fusion protein under the control of the Gal4 upstream activator sequence (UAS) to allow expression of ChR2 in specific tissues and neuronal sub-populations via targetted expression of *GAL4* [14]. To verify that our ChR2 produces light-evoked cellular depolarization in living animals, we first expressed *chr2::yfp* under the control of muscle-specific *GAL4* drivers (*twist*-*GAL4*; *24B*-*GAL4*) and confirmed that there was strong expression of ChR2-YFP in muscles of third-instar larvae. We found that when muscle-specific *GAL4/UAS-ChR2-yfp* animals were raised to the crawling third-instar stage in ATR-containing food they showed strong and rapid responses to illumination (Fig. 1). CO_2_–immobilized larvae contract abruptly upon illumination (Fig. 1A), while freely moving larvae are paralyzed for the duration of the illumination period (Movie S1). Animals that were not fed ATR (Movie S1) or that do not produce ChR2 (data not shown) showed no such paralysis.

In order to determine the temporal specificity and reliability of the muscle response to ChR2, sharp electrodes (10–25 MΩ) were used to record the responses of larval muscles to light pulses (Fig. 1B–D). Illumination generated inward currents that were maintained for the duration of the stimulus with only minor channel inactivation (Fig. 1B, C). The light-dependent response of larval muscles expressing ChR2 was precisely tied in time to repeating light pulses, with precision at the level of 15 milliseconds (ms) or less, and did not significantly diminish with many repeat pulses (Fig. 1B). It was previously shown [11] that pulsed light delivery used in conjunction with ChR2 controls neuronal activity at the level of single action potentials. We found that short light pulses (each pulse 15 ms long) led to the coincident depolarization of larval muscles (Fig. 1D), and even very short pulses (as short as 2 ms) generated detectable depolarization in dissected animals. The timing of the depolarizations correlated precisely with the illumination pulses, demonstrating the tight temporal control of ChR2-mediated photostimulation.

**Figure 2:**
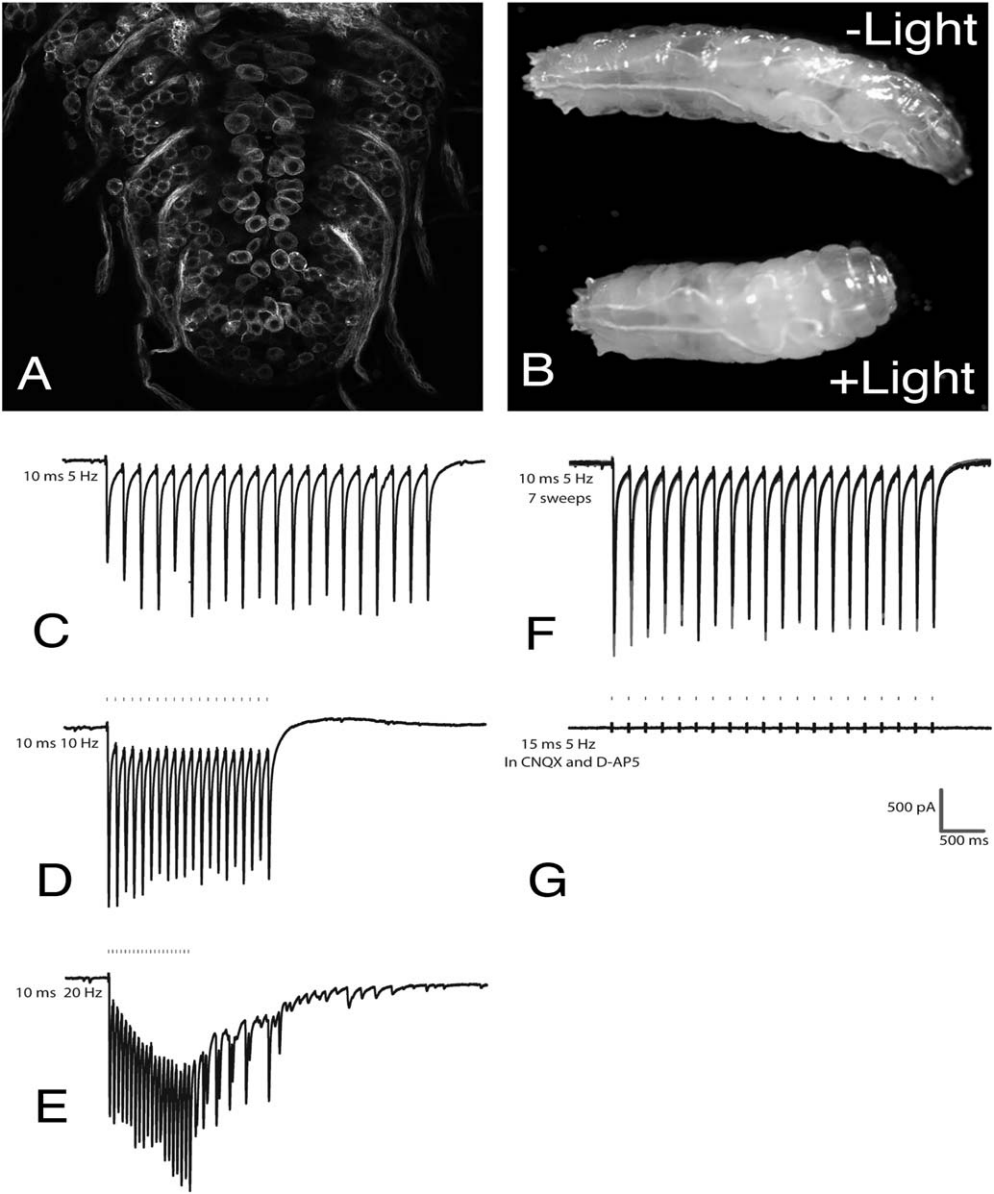
Precise temporal control of motor neuron activity in *Drosophila* larvae. Animals contain one copy of the *D42-GAL4* motor neuron driver and three copies of UAS-*chr2::yfp*. (A) Brain stem of L3 larva producing ChR2-YFP in the cell bodies of motor neurons. (B) A freely moving larva shows rapid body contraction in response to illumination (upper panel prior to illumination, lower panel during illumination) with blue light (0.2 mW/mm^2^). Also see Movie S2. (C–G) One-electrode recordings from muscle 5 of a dissected third instar larva. Short pulses (as short as 2 ms) of blue light (5 mW/mm^2^) generate excitatory junction potentials (EJP's). Precise temporal control of motor neuron activity is demonstrated by varying the frequency of light pulses (lines above traces represent individual light pulses). Trains of EJP's in response to 10 ms light pulses delivered at (C) 5 Hz, (D) 10 Hz and (E) 20 Hz are shown. (F) The responses of the neurons are highly reproducible. This panel shows an overlay of current traces generated by 7 sweeps of 20 light pulses delivered at 5 Hz. (G) Addition of glutamate receptor blockers (CNQX and D-APS) to the preparation blocks light-evoked EJPs.

To test for optical control of neuronal output in *Drosophila*, we expressed ChR2 in motor neurons using the *D42*-*GAL4* driver [15]. *GAL4*-directed production of ChR2-YFP in these neurons revealed that the protein accumulates at the plasma membrane, with a fraction remaining cytoplasmic (Fig. 2A). This suggests that ChR2 is properly localized within neurons in Drosophila for light-driven activation of ChR2 to generate ion conductance across the plasma membrane, and have high levels of ChR2-YFP in the cell bodies and projections of motor neurons. These flies show a strong and immediate contraction in response to illumination (Fig. 2B and Movie S2). This response was completely dependent on ChR2 expression and function, as animals lacking the UAS-*chr2::yfp* gene or deprived of dietary ATR showed no such behavior (data not shown).

### Temporal precision and reliability of ChR2 excitation

Recent studies utilizing ChR2 in *Drosophila* used periods of continuous illumination to generate action potentials [8], [9]. Such a protocol results in variable neuronal activity [8] and does not make use of the precise temporal control demonstrated in cultured mammalian neurons [11]. To examine whether ChR2 expression in *Drosophila* neurons allows us to control neuronal output at the level of single action potentials, we recorded light-evoked excitatory junction potentials (EJPs) in dissected L3 larval muscles in response to short light pulses. Pulses as short as 5 ms were sufficient to generate trains of EJPs at specific frequencies (Fig. 2C–E). At high frequencies (20 Hz and above), the stimulated activity was followed by spontaneous EJPs (Fig. 2E). Overlaying the recordings from 7 independent light-pulse trains demonstrates the uniformity of these responses (Fig. 2F). The addition of CNQX and D-AP5, inhibitors of the glutamate receptor, to the larval preparation completely blocked light-induced EJP's, demonstrating that the light response was due to the activation of glutamatergic motor neurons (Fig. 2G). We also observed that larvae expressing ChR2 in cholinergic neurons (*cha-*GAL4) exhibit rapid head retraction upon illumination (data not shown). These data demonstrate that ChR2-dependent photostimulation allows reproducible and precise temporal control of neuronal activity in Drosophila.

### Light-induced neuronal activity in adult *Drosophila*

**Figure 3:**
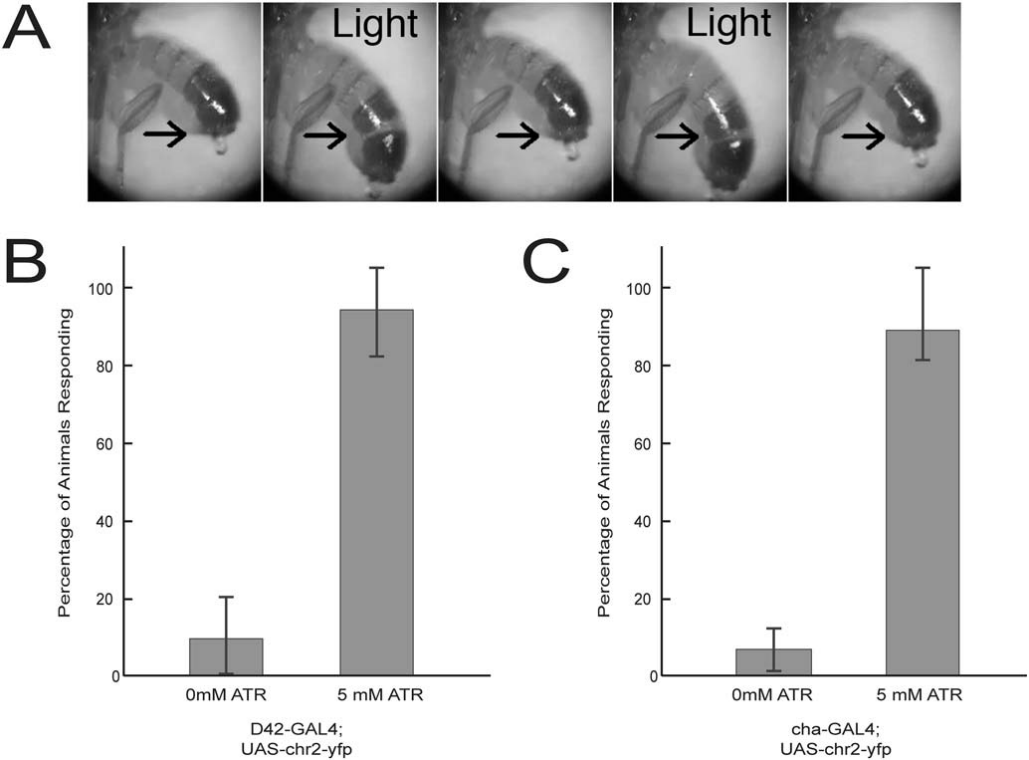
Optical control of behavior in adult flies. (A) CO_2_-anesthetized adult flies that express ChR2 in muscles (*MHC-gs-GAL4*) respond to sustained illumination by contracting abdominal muscles and extending their abdomen. Illumination periods are indicated (‘Light’) and arrows indicate equivalent positions for reference. Also see Movie S3. Adult flies were fed 5 mM ATR and 200 µM RU486 for 3 days prior to assaying. (B) CO_2_-anesthetized adult females expressing ChR2 in motor neurons were assayed for light-induced muscle contractions in leg and abdominal muscles. Animals with one copy of *D42-GAL4* and 3 copies of *UAS-chr2::yfp* display a reproducible light response when they are fed all-trans-retinal (ATR). Little or no response to light was observed with flies that were not fed ATR (0 mM ATR) or that contained only UAS-*chr2::yfp* (not shown). Three groups of 20 flies were assayed. (C) Production of ChR2 in cholinergic neurons produces a light-evoked seizure response in adult flies that are fed ATR (also see Movie S4). Few or no light-evoked responses were observed for animals not raised on ATR-supplemented food. Assays were done in triplicate (n = 20). The seizure response was not observed in animals that contained only one or two copies of UAS-*chr2::yfp* (data not shown). All behaviors were scored blindly with respect to ATR supplementation. Mean±s.d shown for all panels.

In order to explore whether ChR2-mediated photostimulation can be used to informatively probe neuronal circuits that underlie complex behaviors, we first investigated whether ChR2 can be used to manipulate behavior in the adult fly. We expressed ChR2 in adult muscles using the *MHC-gs*-*GAL4* driver, which encodes a *GAL4*/progesterone receptor chimera that becomes active when animals are fed RU486 [16]. We fed ATR (1–5 mM) and RU486 to adult flies for 3 days prior to assaying behavior. We observed robust muscle contraction in response to stimulation, resulting in abdominal extension and protrusion (Fig. 3A and Movie S3).

To examine whether neuronally expressed ChR2 can be used to manipulate adult behavior we examined adult flies in which multiple copies of the *UAS-chr2::yfp* transgene were driven in motor neurons by D42-GAL4. Adults that carried a single copy of *UAS-chr2::yfp* failed to produce a behavioral response, even when animals were fed very high doses of ATR. However, animals carrying three copies of *UAS-chr2::yfp* displayed robust muscle contractions in response to illumination (Fig. 3B). This response depends on ChR2 activity, as flies denied ATR showed little or no response and only 10% (±10%) responded to illumination, while 93% (±12%) of flies raised with supplementary ATR showed abdominal contractions upon illumination.

We also tested the photostimulation of cholinergic neurons using a cha3.1-GAL4 driver to drive four copies of *UAS-chr2::yfp*, and assayed responses in freely moving adults. Stimulation of these flies induced a seizure-like response in approximately 90% of flies show this behavior, and once again the response was dependent on ATR (Fig. 3C and Movie S4). These data suggest show that stimulation of the central nervous system may be possible when strongly expressing *UAS-chr2::yfp* is at high levels.

### Light-mediated control of neuronal circuits in adult flies

We next set out to use ChR2 to explore the neuronal basis of the innate escape responses that *Drosophila melanogaster* adults exhibit in response to various stimuli, including exposure to puffs of odorants or a physical jolt. The *acj6* gene is functional in at least some of the neurons important for the manifestation of escape responses, since mutations in the *acj6* gene reduce the flies' apparent escape response to either an aversive odorant puff or a physical jolt [17], [18], [19]. The *acj6* gene encodes a POU domain transcription factor that is expressed in a subset of primary and secondary olfactory neurons, and is necessary for the proper wiring and development of the olfactory circuitry [20], [21]. The *acj6* gene is also expressed in the visual system, central brain, and ventral nerve cord [22].

**Figure 4:**
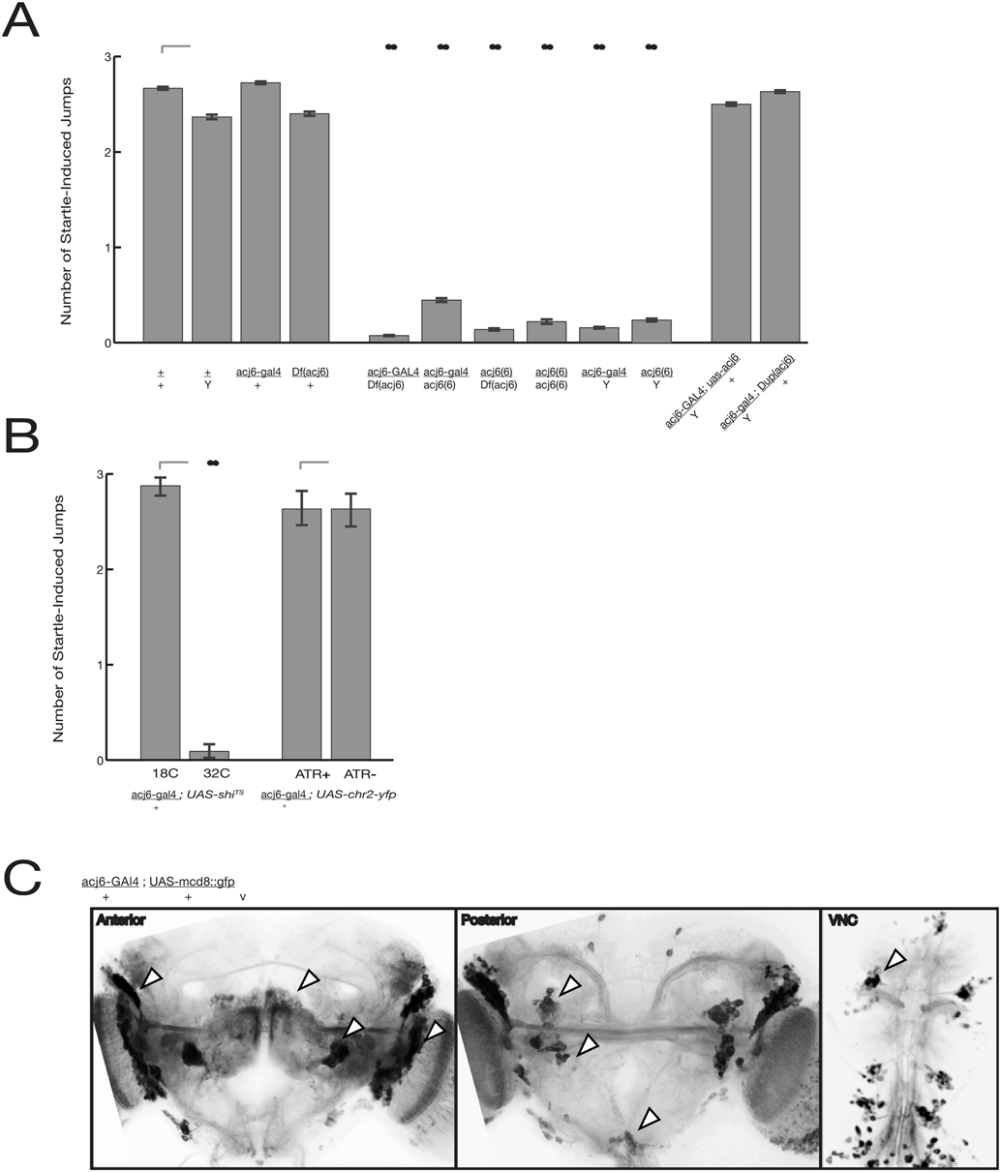
Genetic and physiological disruption of the escape response. (A) Flies were tested for a jump response within 3 s of bumping their vial on the table; the average number of jumps over three trials is shown. Hemizygous mutant males (*acj6*-*GAL4*/Y) show an attenuated startle response. Data shown as mean±s.e. (B) Silencing *acj6-GAL4* expressing neurons with the temperature-sensitive dynamin allele *shibire* ^TS^ in *acj6* neurons recapitulates the *acj6* mutant phenotype, but expressing ChR2 with or without ATR generates no deficit. (C) *acj6-GAL4* expression as revealed by *UAS*-mcd8::GFP in the anterior and posterior brain, as well as the VNC. Arrowheads mark notable cell clusters. For all figures, the double-asterix shows significance at p<0.001 vs. the relevant control.

The existence of a GAL4 enhancer trap inserted at the *acj6* locus [23] potentially provides the tool needed to express *UAS-ChR2-yfp* in the *acj6* neurons necessary for escape responses. Any given enhancer trap may not capture the full expression pattern of a gene, so it was necessary to characterize the expression pattern of *acj6-GAL4*. In *acj6-GAL4* bearing flies, Gal4 is produced in primary and secondary olfactory neurons as well as in a subset of neurons in the central brain and ventral cord (Fig. 4C) [24]. Endogenous *acj6* expression has been reported in the central complex of the brain, which is implicated in the coordination of locomotor behavior [22], reviewed in [25], but we did not detect *acj6-GAL4* expression in this region. Thus *acj6-GAL4* may not recapitulate the entire *acj6* expression pattern, or may not truly express in neurons innervating the central complex.

We tested the phenotype of the *acj6-GAL4* allele as a way of ascertaining whether this allele influences neurons involved in the startle responses. We found that the insertion of GAL4 into the *acj6* gene in *acj6-GAL4* generates an *acj6* mutant phenotype. Using a simple startle assay, in which a vial containing a single fly is tapped on a hard surface to induce a jump and brief flight, we reproduced the findings that a deletion of *acj6 [df(acj6)]* and a null allele of *acj6 [acj6^6^]* both severely reduce the startle response ([19]; Fig. 4A). Most importantly *acj6-GAL4* exhibits as severe a reduction of the startle response as the null mutations (acj6-Gal4/Y vs. acj6^6^/Y, p>0.4; acj6-Gal4/Df(acj6) vs. acj6^6^/Df(acj6), p>0.4; acj6-Gal4/acj6^6^ vs. acj6^6^/acj6^6^, p>0.2). That these effects are due to the lesions in the *acj6* gene was shown by the fact that restoring *acj6* expression using a gene duplication or by driving expression of an *UAS*-*acj6* transgene with *acj6-GAL4* rescued the startle response.

The next question was whether the neurons where *acj6-GAL4* is expressed are the locations of *acj6* function necessary for the startle response. We confirmed that the startle response requires the activity of these *acj6-GAL4* neurons by inhibiting these neurons in other ways than the GAL4 insertion. When we expressed the temperature-sensitive dynamin allele *shibire* ^TS^ under the control of *acj6-GAL4*, the startle response was normal in heterozygous flies at permissive temperatures, but completely abolished at restrictive temperatures (p<0.001 vs. permissive temperatures; Fig. 4B). Taken together, our findings establish that both the function of the *acj6* locus, and the activity of *acj6*-expressing neurons, are necessary for the wild-type startle response.

**Figure 5:**
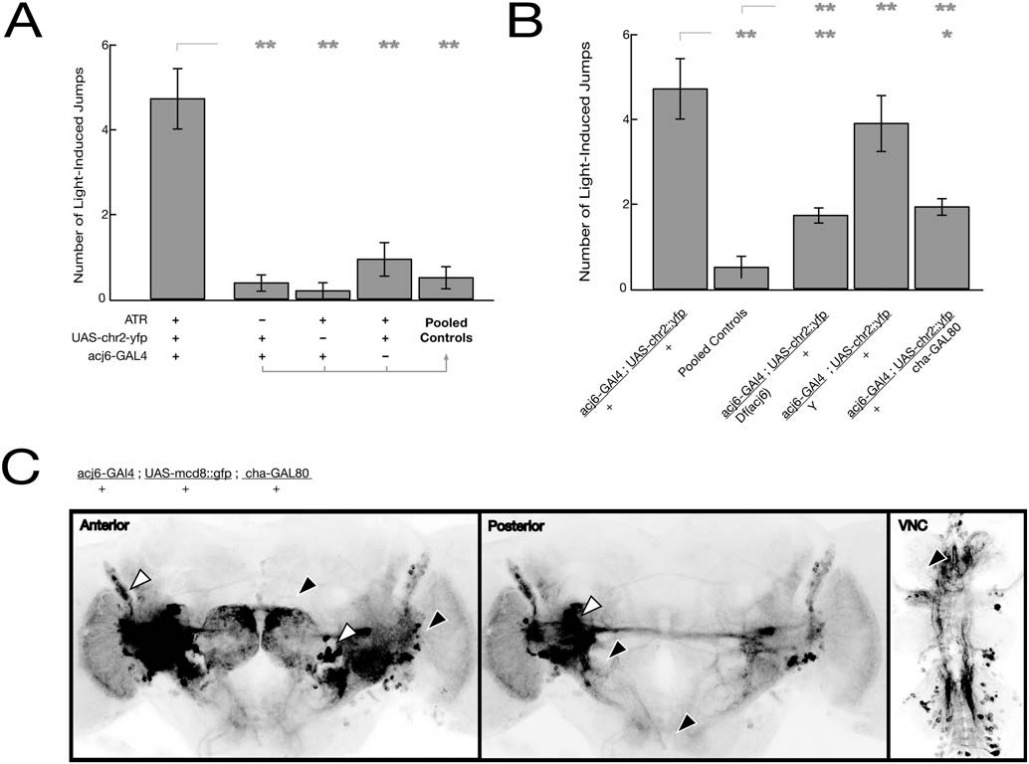
Induction of a synthetic escape response. (A) ChR2-mediated excitation of *acj6-GAL4* expressing neurons in female flies; number of jumps pooled over three 15 s illumination periods. Photoexcitation of *acj6* neurons is sufficient to induce a startle response. Pooled controls are all flies lacking ATR or one transgene. Data shown as mean±s.d. (B) ChR2-mediated excitation of *acj6* neurons in mutant flies, as well as photoexcitation restricted to non-cholinergic *acj6* neurons by the cha-*GAL80* transgene. The light-induced jump response is attenuated in females held over deficiency, but not in hemizygous male flies. Data shown as mean±s.d (C) *acj6-GAL4* expression in non-cholinergic neurons, with expression limited by the *cha3.1-GAL80* transgene. White arrowheads mark cell clusters retained in *cha-GAL80* flies, as compared to Fig. 4C, and black arrowheads mark those largely absent; see text for details. Asterix denotes p<0.05 vs. the relevant control, double-asterix denotes p<0.001.

Based on these findings we used *acj6-GAL4*-driven *UAS-ChR2-yfp* expression to ask whether light-induced activation of *acj6-GAL4* neurons was sufficient to generate a synthetic escape response. Phenotypically, *acj6^+^ f*emale flies expressing ChR2 in *acj6* neurons respond to illumination in the absence of any physical jolt with a reproducible escape or startle response, including repetitive jumping and flight (see Movie S5). This response depended on ChR2-mediated excitation of the *acj6* neurons, as it required supplementary ATR (Figure 4E, p<0.001). Dietary ATR was not sufficient to elicit the light-induced jump response in flies producing no ChR2, i.e. flies carrying only the *acj6-GAL4* or only the *UAS-chr2::yfp* transgene (Fig. 5A).

We tested whether *acj6* function was necessary for the light-induced jump response by testing female flies carrying *acj6-GAL4* over a deficiency spanning *acj6*. Photoexcitation of these presumably compromised neurons generated an attenuated response that was still readily distinguishable from the control levels without ChR2 expression or without ATR (Fig. 5B; p<0.001 vs. *acj6-GAL4/+; UAS-chr2::yfp*+ATR; p<0.001 vs. pooled controls lacking either one transgene or ATR). Therefore the *acj6* mutation in these females either does not abolish the function of all *acj6* neurons, or does not prevent them from activating downstream neurons in response to photostimulation. We also tested male flies, having previously shown that the hemizygous *acj6-GAL4* insertion mimics a null allele of *acj6* in the bang assay. Photoexcitation in these males still generated a robust light-induced jump response (p>0.1 vs. *acj6-GAL4/+; UAS-chr2::yfp*+ATR; p<0.001 vs. pooled controls). Importantly, the endogenous startle response – as tested by the bang assay - remains intact when ChR2 is expressed in *acj6*-expressing neurons, demonstrating that neuronal function is not impaired by over-production of *GAL4* or of the ChR2 channel protein (Fig. 4A, p>0.6).

From these experiments it is clear that *acj6* function contributes to the light-activated jump response. Furthermore, photoexcitation is sufficient to induce an escape response from flies that appear, by traditional mutant analysis, incapable of such a response. We tested whether the light-induced jump response depended on ChR2 function in cholinergic *acj6* neurons, or could be induced by the (largely central) non-cholinergic acj6 neurons. Cholinergic neurons known to express *acj6* include olfactory sensory neurons (OSNs) in the antennae and maxillary palp and a subset of *acj6*-expressing neurons in the central nervous system (CNS) [19] We used the *cha-GAL80* transgene [26] to specifically inhibit the activity of *GAL4* in cholinergic neurons, thus preventing activation of ChR2 there. Females that carried *cha-GAL80* in addition to *acj6-GAL4* and UAS-*chr2::yfp* had a reduced startle response compared to animals without *cha-GAL80* (Fig 5B; p<0.001). The response remained significantly greater than pooled negative controls (p<0.04), suggesting that GAL80 did not completely suppress the activity of GAL4 or that excitation of cholinergic *acj6* neurons facilitates the light-activated jump response but is not essential.

The olfactory neurons known to express *acj6-GAL4* are cholinergic [27], so the ongoing effects of ChR2 despite *cha-Gal80* suggests that activation of non-cholinergic central neurons may contribute to the synthetic jump response . To examine this possibility more closely, we compared *acj6-GAL4* expression with and without *cha-GAL80* repression, using a UAS-mcd8::GFP reporter (Fig. 5C). *cha-GAL80* causes the loss of GFP production from several classes of putatively cholinergic neurons: projection neurons innervating the lateral horn, possible gustatory interneurons that project from the SOG to the deuterocerebrum, and several laminar neurons of the optic lobe. In the ventral nerve cord, thoracic production of GFP was largely abolished, so these neurons were presumably also cholinergic. Neuronal populations continuing to produce GFP are presumable non-cholinergic, and can be expected to express ChR2 in these experiments. These include interneurons lateral to and likely innervating the antennal lobe, as well as very minor expression in projection neurons, which may be the GABA-ergic (Wilson and Laurent, 2005). Other cell populations are less well described, but appear to innervate the optic lobes and lateral horn; some descending tracts also remain, while the majority of thoracic expression is lost. Although it is difficult to determine which of these neurons is most imortant in this assay, we conclude that photoexcitation of some of these central neurons may be sufficient to induce the escape response.

Acj6 has previously been known to be necessary for the chemosensory jump [20], [21] and the startle response to a physical jolt [19]. Here we have demonstrated that the activity of acj6 neurons is necessary for an escape in response to a physical jolt, and that photoexcitation is sufficient to generate a synthetic escape response. We conclude that *acj6* neurons in the central brain may mediate a general escape response circuit, which is triggered by a variety of alarm signals. Further experiments will be necessary to relate this circuit to the giant fiber circuit traditionally associated with the escape response.

## Discussion

Historically, activation-based analyses of the neural basis of behavior have been limited to electrode manipulations that involve non-specific stimulation of clustered cells or direct stimulation of single cells. Genetic advances in *Drosophila* provide for targeted expression of controllable molecules in genetically defined neurons, allowing regulated inhibition of neuronal activity [28], [29]. Approaches based on neuronal stimulation in *Drosophila* and other genetically tractable organisms have often been limited by a lack of efficient means for activating dispersed neurons in behaving animals.

Photostimulation of genetically specified neural circuits using the directed expression of the ChR2 channel rhodopsin can be effectively used to regulate neuronal output. The stimulation of aminergic neurons in *Drosophila* larvae, for example, was previously shown to induce aversive and appetitive reinforcement [8]. More recently, ChR2 was used to manipulate odorant neurons in adult flies [9]. We demonstrate here that ChR2 stimulation offers temporal control of neural activity with millisecond precision over a wide range of frequencies, and can be used to manipulate both larval and adult behaviors. Such tight control of neural activity is a prerequisite if such photostimulation is to be used to mimic environmental stimuli, or for activation-based screens to identify the neural circuitry underlying innate behaviors.

### Triggering an escape response using photostimulation of discrete, dispersed neurons

To apply ChR2 to behaviorally significant circuitry, we manipulated neurons necessary for the adult startle response. The startle response is disrupted by mutations in the *acj6* transcription factor. *acj6* expression is found in neurons at multiple points along sensory pathways, and is required for their proper specification and wiring [20], [21], [27]. Because *acj6* appears to regulate the physiology of a subset of these neurons, we hypothesized that activity of neurons expressing *acj6* would be necessary and sufficient for the jump behavior that does not occur in *acj6* mutants.

We have shown that the activity of *acj6* neurons is necessary for a physical startle response response, and that silencing these neurons recapitulates the mutant phenotype. Stimulation of these neurons through ChR2 photostimulation induces a synthetic jump response that mimics the wild-type startle response. This suggests that the escape response associated with the *acj6* phenotype is in fact mediated by *acj6*-expressing neurons, whose activity is necessary and sufficient for this behavior.

### Role of *acj6* in facilitating the startle response to light-activated ChR2

Photostimulation is capable of inducing a wild-type response in males hemizygous for the *acj6-GAL4* insertion, and induces an attenuated response of females carrying *acj6-GAL4* in trans to an *acj6* deficiency. The more robust effect in males may be attributable to dosage compensation, which could increase transcription of *acj6*, to a level sufficient for photoexcitation but not sufficient for wild-type behavior, or of *gal4*, to a level sufficient to overcome the *acj6-GAL4* mutation. Several roles for *acj6* are compatible with this effect: the *acj6-GAL4* mutation may reduce or alter activity in some neurons, such that photoexcitation can induce a jump response but normal stimuli cannot. Alternatively, the *acj6-GAL4* mutation may alter connectivity, as reported for *acj6* mutation in photoreceptors and projection neurons [20], [21], [22].

Preventing ChR2 expression in cholinergic neurons with *cha-Gal80* partially eliminates the synthetic jump response. The remaining activity may be due to residual photoexcitation of some cholinergic *acj6* neurons, or ChR2 function in non-cholinergic *acj6* neurons - largely limited to CNS interneurons - may be sufficient to drive the synthetic jump response. The latter possibility would mean that a subset of *acj6* neurons comprises a circuit whose activity is necessary and sufficient for the escape response.

Several drawbacks to photostimulation remain. In most experiments, illumination levels are quite high, and reliable photostimulation of the CNS is limited by the ability to provide enough light to penetrate the cuticle without overheating the animal. More detailed analyses, such as eliciting a behavior through the excitation of single neurons, remains elusive but may be resolved by MARCM techniques or development of a library of GAL80 repressors.

Photostimulation of genetically identified neural populations is a powerful tool for inhibiting or inducing behaviors in an awake animal. Directed stimulation of neuronal populations can be used to identify novel circuitries underlying other innate behaviors. Because these experiments rely upon genetic targeting, they will also reveal genes and enhancers whose expression is limited to specific neurons underlying a behavioral circuit and may therefore play a role in their formation or physiology.

Photostimulation may also be used to manipulate neuronal activity in the behaving animal to understand how information is processed during behavior, and reveal the modulation of sensation, integration, and motor output during behavior. When integrated with genetic and physiological analyses, these approaches will contribute powerfully to revealing the genetic and neural basis of behavior.

## Methods

### Plasmids

Gateway *att*B recombination sequences were added to the ends of the *chr2::yfp* fusion gene^9^ by PCR-amplification with primers 5′-GGGGACAAGTTTGT ACAAAAAAGCAGGCTATGGATTATGGAGGCGCCC-3′ and 5′- GGGGACCACTT TGTACAAGAAAGCTGGGTTTACTTGTACAGCTCGTCCAT-3′. The PCR product was first cloned into pDONR™201 vector (Invitrogen) using BP Clonase™ (Invitrogen) and subsequently into the pTWC fly transformation vector (Drosophila Genomics Resource Center) using LR Clonase™ (Invitrogen). The pTWC vector contains the UASt promoter [14] and a C-terminal eCFP tag. The eCFP tag is not expressed due to a stop codon at the end of *chr2::yfp*.

### Fly lines

Germline transformations of *yw* flies with pTWC-*chr2::yfp* was performed by standard techniques. We used two *UAS*-*chr2::yfp* transgenic lines with homozygous viable P element insertions on the third chromosome in combination with the Gal4 drivers *twist-GAL4*, 24B-*GAL4*, D42-*GAL4*, *cha-GAL4* and *acj6-GAL4* [24] to express ChR2. We generated the stocks *yw*;; D42-*GAL4*, *UAS*-*chr2::yfp*/TM3 and *yw*;; 2×*UAS*-*chr2::yfp* to obtain larvae and adults containing D42-*GAL4* in combination with one, two or three copies of *UAS*-*chr2::yfp*. We also generated the stock *yw*; *cha-GAL4*/Cyo; 2×*UAS*-*chr2::yfp* to study adults expressing high levels of ChR2 in cholinergic neurons.

### Electrophysiology

Larvae were raised on fly food supplemented with all-trans retinal (Sigma, 100-200 nmol added daily on top of vials containing ∼5 ml of standard medium) until the third instar stage, then dissected in HL3 buffer [30]. Intracellular recordings were from muscle 5 (for experiments probing motor neuron expression of ChR2) and 6 (for experiments testing muscle expression of ChR2) using microelectrodes with a resistance at 5 to 10 mOm filled with HL3 buffer. Resting membrane potentials were typically between −40 mV and −60 mV. Recordings were performed using an Axon Multiclamp 700B (Molecular Devices Corporation). Light was delivered with a Sutter Lambda DG-4

rapid-wavelength switcher (Chroma HQ470/40× excitation filter), and a 300-W Xenon lamp giving rise to ∼10 mW/mm2 at the focus with 5× immersion objective. For details see.

### Behavior

Larvae were raised to the third instar stage on fly food supplemented with all-trans retinal (Sigma, 100–200 nmol added daily on top of vials containing ∼5 ml of standard medium) before testing their response to short (1–2 second) illumination periods using blue light at an intensity of about 0.2 mW/mm^2^. Imaging and illumination were performed on a Leica MZFLIII fluorescent microscope.

Adults expressing ChR2 in motor neurons (yw;: D42-*gal4*, UAS-*chr2::yfp*/2×UAS-*chr2::yfp*) and cholinergic neurons (yw; *cha-gal4*/Cyo; 2×UAS-*chr2::yfp*) were raised on 5 mM all-trans retinal for 2–3 days prior to assaying. Individual flies that express ChR2 in motor neurons were assayed by anesthetizing them with CO_2_ for 30 seconds before evaluating abdominal contractions in response to light. Behavior was assayed alongside control flies (flies with the same genotype raised in the absence of retinal) and scored blindly. Individual flies that express ChR2 in cholinergic neurons were assayed by placing them in a 96-well dish (mostly filled with 1% agar), allowing them to recover for one minute, and then evaluating their response to light (1–3 second illumination at 0.2 mW/mm^2^) over a 20 second period. Behavior was scored blindly alongside animals that were not fed all-trans retinal.

For experiments with *acj6*-*GAL4*, larvae were raised until eclosion on standard fly food supplemented daily with ∼60 µL 1 mM ATR, and housed collectively with a similar supplement until testing at 7–12 days. Flies were tested as yw,*acj6*-GAL4/yw;;2×UAS-CHR2. Controls were outcrossed to the strain Canton-S A. The response of an individual fly was tested under 15 seconds of bright light (combining ∼0.2 mW/mm^2^ blue light with ∼1 mW/mm^2^ white light), repeated three times. The total number of jumps was scored blindly, summed across the three trials, and this sum transformed as x = √x+1 for analysis. The innate startle response was tested by tapping a vial with a single fly onto a hard surface; a jump within three seconds was scored as a success. This procedure was repeated three times, and reported as the average number of jumps in three trials. *Shibire* flies were tested as *acj6*-GAL4/uas-shi^TS^; uas-shi^TS^, and were raised at 18°C until one hour before testing [29]. N>18 for all trials.

## Supporting Information

Movie S1 Larvae expressing ChR2 in muscles under the control of twist-GAL4 and 24B-GAL4. Illumination causes paralysis in an ATR-dependent manner.
(0.56 MB MOV)

Movie S2 Larvae expressing ChR2 in motor neurons with the D42-GAL4 driver show a full-body contraction upon illumination.
(0.70 MB MOV)

Movie S3 Adult flies expressing ChR2 in muscle under the MHC-gs-GAL4 driver. Photostimulation results in robust muscle contraction, resulting in abdominal extension and protrusion.
(0.30 MB MOV)

Movie S4 Adult flies expressing ChR2 in cholinergic neurons under cha-GAL4. Photostimulation results in a seizure-like response, which is dependent on supplementary ATR.
(1.32 MB MOV)

Movie S5 Adult female flies expressing ChR2 under the acj6-GAL4 driver. Photostimulation induces a startle-like jump response, dependent on supplementary ATR.
(2.72 MB MOV)
